# Who Moves In Matters: Move-in Patterns, Household Headship, & Financial Strain Among Latino Adult Child Caregivers

**DOI:** 10.1093/geroni/igaf122.3328

**Published:** 2025-12-31

**Authors:** Elizabeth Vásquez, Jacqueline Angel, Sunshine Rote, Phillip Cantu, Anna Bokun

**Affiliations:** University at Albany (SUNY), Albany, New York, United States; The University of Texas at Austin, Austin, Texas, United States; University of Louisville, Louisville, Kentucky, United States; UTMB Health, The University of Texas Medical Branch, Galveston, Texas, United States; The University of Texas at Austin, Boston, Massachusetts, United States

## Abstract

While research shows increasing prevalence of multigenerational households, we know little about how different pathways to co-residence affect caregiver well-being. Using the Hispanic Established Populations for the Epidemiologic Study of the Elderly (2010-11), we examine how move-in scenarios and household structure influence financial strain among 659 Mexican-American adult child caregivers and their older parents (80+). Preliminary findings show that financial strain varies by move-in scenario. Adult children who moved in with their parent to provide care experience the highest rates of financial strain (22.2%), followed by adult children whose parents moved in with them for care (19.4%). Homeownership is a key predictor, with financial strain reaching 35.7% among caregivers who moved into a parent-owned home to provide care. Parents with dementia are more likely to move in with their adult children (64.5% vs. 44.4% when children move in), suggesting cognitive decline drives household restructuring. Among adult children who moved in to provide care, only 14.8% are household heads, compared to 57.1% of those who moved in for general reasons. This housing transition correlates with increased caregiving intensity (8.0-8.1 ADL/IADL hours per day) vs. 2.6-3.4 hours for caregivers who moved in for their own housing needs, and 6.2-6.3 hours for caregivers who co-reside without discerning move-in reasons.These findings highlight the need for policies that consider both direction and motivation for household moves when targeting support for caregiving families. Future research incorporating American Community Survey data will examine broader patterns of caregiving-related mobility and how these patterns compare across other populations.

